# Colorectal Tumors from APC*I1307K Carriers Principally Harbor Somatic *APC* Mutations outside the A8 Tract

**DOI:** 10.1371/journal.pone.0084498

**Published:** 2014-01-09

**Authors:** Peter Zauber, Timothy Bishop, Claire Taylor, Marlene Sabbath-Solitare, Stephen Marotta, Ian Tomlinson

**Affiliations:** 1 Department of Medicine, Saint Barnabas Medical Center, Livingston, New Jersey, United States of America; 2 Section of Epidemiology and Biostatistics, Leeds Institute of Molecular Medicine, University of Leeds, Leeds, United Kingdom; 3 Cancer Research UK Genomics Facility, Leeds Institute of Molecular Medicine, University of Leeds, Leeds, United Kingdom; 4 Department of Pathology, Saint Barnabas Medical Center, Livingston, New Jersey, United States of America; 5 Molecular and Population Genetics, Wellcome Trust Centre for Human Genetics, Oxford, United Kingdom; IFOM, Fondazione Istituto FIRC di Oncologia Molecolare, Italy

## Abstract

**Purpose:**

APC*I1307K (c.3920T>A) is an inherited variant associated with colorectal tumour risk found almost exclusively in those of Ashkenazi Jewish ancestry. A single nucleotide substitution creates an oligo-adenine tract (A8) that appears to be inherently prone to further mis-pairing and slippage. The reported multiple tumor phenotype of carriers is not easily reconciled with molecular and population genetics data. We postulated that some c.3920T>A carriers with multiple adenomas have other unidentified APC germ line or somatic mutations.

**Methods:**

DNA from 82 colonic tumours and accompanying normal tissue collected from 29 carriers with multiple colorectal tumors was directly sequenced between codons 716 and 1604. We also assessed APC gene loss of heterozygosity.

**Results:**

One patient (3.4%) was found to have an additional APC germ line mutation. Twenty-five of the tumours showed no significant somatic molecular change, 36 showed one change, 20 showed two, and one tumour showed more than 2 changes. Our data suggest a correlation between advancing histology and fewer beta-catenin binding sites remaining in the mutant proteins.

**Conclusions:**

There were no other common germ line variants identified within the region of the APC gene examined, suggesting that any effect from this region on tumour production is attributable to the c.3920T>A allele. Our findings further suggest the only somatic genetic change clearly attributable to the c.3920T>A mutation is the c.3924_3925insA.

## Introduction

Both germ line and somatic mutations within the *Adenomatous Polyposis Coli* (*APC*) gene are well established as important in colorectal carcinogenesis [1]–[3]. The syndrome Familial Adenomatous Polyposis (FAP), associated with the development of hundreds to thousands of adenomatous polyps during teenage years, is due to dominant germ line mutations in the *APC* gene. A variant, attenuated FAP (AFAP) is characterised by tens of adenomas and results from germline mutations in specific regions of the *APC* gene (exons 1–3, exon 9, and the second half of exon 15). Further, most sporadic colorectal cancers acquire somatic *APC* mutations and the pattern of somatic genetic changes is consistent with *APC* being a tumor suppressor gene [1].

Almost all germ line *APC* gene mutations involved in FAP result in a truncated or absent protein. However, a novel mechanism of tumorigenesis involving site-specific hypermutability in the *APC* gene around codon 1307 (APC*I1307K or c.3920T>A) has been described [4]. This germ line variant results in an adenine replacing a thymine and creating an oligo-adenine (A8) tract that appears to be inherently prone to further somatic mis-pairing and slippage during DNA replication, thereby creating a frameshift change. The c.3920T>A mutation is found almost exclusively in those of Ashkenazi Jewish ancestry [4]–[5]. Although the mutation was reported to be responsible for a phenotype of multiple colorectal adenomas and carcinoma, other reports have indicated the number of tumours to be limited, usually less than 10 [6]–[7].

It is difficult to reconcile the reported multiple tumour phenotype of c.3920T>A carriers with A8 slippage causing an AFAP-like phenotype, for four main reasons. First, the frequency of this polymorphism in Ashkenazim is relatively high (∼6% carrier frequency). It seems likely that a dominant allele predisposing to multiple early-onset neoplasms would be selectively disadvantageous and would not reach such a high frequency [8]. Second, supporting this contention, the relative risk of colorectal cancer in patients with c.3920T>A is only about 1.5, whereas the cancer risk in AFAP syndromes is several-fold higher. Third, many tumours from c.3920T>A carriers do not carry mutations resulting from slippage of the c.3920T>A allele. In the original report of this mutation, DNA samples from 23 colorectal neoplasms were sequenced between codons 1296 and 1322 [4]. Although 11 of the 23 tumours had acquired somatic *APC* mutations, all in the c.3920T>A allele, only 6 of these tumours (26%) had acquired a somatic mutation by slippage of the A8 tract. In another similar study, a minority (53 of 127, or 42%) of tumours had mutations involving the A8 tract [9]. We ourselves previously reported that only 10 of 18 (55.6%) tumours from c.3920T>A carriers had somatic mutations within the A8 tract [10].

Fourth, *APC* requires ‘two hits’ to inactivate its tumour suppressor activity. In adenomatous polyps, a ‘second hit’ by loss of heterozgyosity (LOH) tends to occur when the germ line mutation is in the region between the first and second beta-catenin degradation repeats (codons 1285–1378) [11]. Since c.3920T>A is in this region, it would be expected that frame shift within the A8 tract might be accompanied by LOH of the germ line wild type allele as the second hit. We subsequently reported a larger series of 120 tumors from patients with the c.3920T>A mutation, in which 40 (33%) had acquired a somatic adenine insertion, c.3924_3925insA, within the A8 tract, thus creating a frame shift and a STOP codon. Twelve (30%) of these 40 tumors demonstrated *APC* LOH , in each case involving the germ line wild-type allele (6). Similar results were shown by others [9]. Tumours that did not show slippage of the A8 tract, and therefore retained the germ line missense variant, showed very little evidence of LOH. These data strongly suggest that the tumours without A8 slippage did not arise as a direct result of the functional consequences of the c.3920T>A mutation.

The combined evidence suggests that c.3920T>A patients might not typically have a multiple adenoma phenotype, and that those with multiple tumours might be a highly selected group of c.3920T>A carriers. Potential explanations include: 1) another germ line *APC* mutation in linkage disequilibrium with c.3920T>A in some or all Ashkenazi patients with multiple colorectal neoplasms, 2) a tendency for the c.3920T>A allele to undergo slippage in some patients more than in others, and 3) patient study groups have had an additional, uncharacterized tendency to form colorectal tumours.

Detailed examination of patterns of germ line and somatic changes in the *APC* gene within this cohort may provide further evidence regarding the effect of the c.3920T>A variant in colorectal carcinogenesis. Therefore, we have analyzed an extended region of the *APC* gene in DNA extracted from tumours removed from Ashkenazim carrying c.3920T>A, and for whom DNA was available from multiple colorectal tumours. This study extends both the number of tumours and the size of the genetic region examined compared to previous studies.

## Materials and Methods

### Patients

Patients undergoing colonoscopy at two busy endoscopy suites between January 2000 and March 2003 were accrued if they had any prior or current colorectal tumours. With the permission of their colonoscopists, they were contacted to verify Ashkenazi ancestry and invited to participate in a study designed to assess germ line variation in cancer-predisposing genes; exploration of c.3920T>A was a major feature of the study. Informed consent was obtained in writing from each participant. Personal and family history was obtained by telephone. The Saint Barnabas Medical Center Institutional Review Board approved the content and ethics of the study. All patients provided signed consent.

For those reporting Ashkenazi ancestry, normal tissue was obtained to determine c.3920T>A status; this was either banked normal colon tissue when available or peripheral blood in the absence of normal tissue. Testing for carrier status was conducted using the PCR-based methods described below. Information on prior total colonic pathology burden was obtained from hospital pathology records, the colonoscopists' office records and personal interviews. For logistical reasons, no systematic attempt was made to locate all outside prior materials, although for a few participants such efforts were made if local material was limited and if outside material was readily available. For those persons shown to be carrying the c.3920T>A variant, efforts were made to locate all pathology material previously submitted to our hospital Pathology Department, as well as pathology reports for all tumours found by other centers. The frequency of carriers of c.3920T>A in the population was previously reported as 12.6% of all those reporting Ashkenazi ancestry and having a colonoscopy [10]. For this study, all patients were carriers of the c.3920T>A mutation and, for most, had at least two colorectal tumours. One clinical pathologist reviewed all histological slides and indicated the areas for molecular study. Criteria for differentiation of adenomas followed the World Health Organization criteria with respect to villous component: tubular adenomas, <20%; tubulovillous adenomas, 20–80%; and villous adenomas, >80%. There is an overlap of patients between this study and our previous report [10], with 4 persons being included in both studies; however, only 4 tumours from 3 patients are in common between the two reports (2 tubular adenomas, 1 tubulovillous adenoma and one cancer). The patients whose tumours constitute this study were also included in our genetic epidemiological paper [6].

### PCR Analysis to Determine *c.3920T>A* Carrier Status

Initial studies for the c.3920T>A mutation status were performed on DNA from peripheral blood or paraffin-embedded normal tissue by one investigator (SM) using the Puregene DNA Isolation Kit (Gentra Systems, Minneapolis, MN, USA) and the QIAamp DNA Mini-Kit (Qiagen, Valencia, CA, USA) respectively. For evaluation of somatic changes, neoplastic tissue with as little normal tissue admixed as possible was identified from sections stained with haematoxylin and eosin. The relevant area from the next, unstained section was isolated manually using a blade under a dissection microscope. A microdissection instrument was not used. The paraffin wax was removed by xylene and ethanol washes. Cellular material was lysed in a proteinase K+ buffer solution. Extracted DNA was amplified using the polymerase chain reaction (PCR), and post PCR product was purified using the QIAquick PCR Purification Kit (Qiagen, Valcencia, CA, USA).

A segment of the *APC* gene consisting of 262 bases was amplified using the primer set 5′-CCA ATA TGT TTT TCA AGA TGT AGT TC-3′ and 5′-AA TTC AAC AGC TTT GTG CCT-3′. Sequencing of this amplicon effectively screens codons 1277 through 1348. Reactions were carried out in 50 µl volumes using Qiagen buffer and Taq polymerase (Qiagen, Valencia, CA, USA), 0.5× Q-solution (Qiagen, Valencia, CA, USA), 200 uM each dNTP, 2.5 mM MgCl_2_, and 50 pmol of each primer. Post PCR product was purified using the QIAquick PCR Purification Kit (Qiagen, Valencia, CA, USA) and an aliquot was run on an agarose gel to determine quality.

The purified product was sequenced using the Big Dye Terminator sequencing chemistry (Applied Biosystems, Foster City, CA, USA) and 4 picomoles of either sense or antisense primer. Cycle sequence conditions were: 25 cycles of 96°C for 10 seconds, 50°C for 5 seconds and 60°C for 4 minutes. Unincorporated nucleotides were cleaned using AutoSeq G-50 Microspin columns (Amersham Pharmacia Biotech, Piscataway, NJ, USA). Electrophoresis was performed on an ABI Prism 377 DNA Sequencer (Applied Biosystems, Foster City, CA, USA) using a 5% Long Ranger denaturing acrylamide gel (Biowhittaker Molecular Applications, Rockland, ME, USA). Data analysis utilized DNA Sequencing Analysis Software (Applied Biosystems, Foster City, CA, USA) followed by visual inspection of the electropherograms.

### PCR analysis for *APC* gene Loss of Heterozygosity

Loss of Heterozygosity (LOH) of the *APC* gene was determined through the PCR amplification of a CA repeat microsatellite marker within the D5S346 locus of the *DP1* gene. PCR reactions were carried out in 30 ul volumes using Applied Biosystems reagents (Roche Molecular Systems, Inc., Branchburg, NJ). Four picomoles of each primer and a 1.5 mM MgCl_2_ concentration was used in the PCR reactions. The primers had a 5′-6FAM label on the sense strand and a 5′GTGTCTT tail on the anti-sense strand. Cycling conditions were as follows: 6 minutes denaturation at 94°C, followed by 35 cycles of a 30 second denaturation at 94°C, 25 second annealing at 55°C, and a 50 second elongation at 72°C, with a final 30 minute extension at 72°C. PCR products were loaded onto a 5% Long Ranger acrylamide gel (Biowhittaker Molecular Applications, Rockland, ME, USA) containing 6M urea and analyzed on an ABI Prism 377 DNA Sequencer with GeneScan collection software (PE Applied Biosystems, Foster City, CA).

The D5S346 alleles are designated A1 through A15. For all LOH analysis neoplastic tissue was evaluated simultaneously with normal tissue or blood DNA from the same patient. The ratio of the height of the allele band intensities for the two alleles from neoplastic tissue was divided by the corresponding ratio for the normal mucosal tissue. LOH was defined operationally as a resultant ratio of less than or equal to 0.5. We previously reported complete linkage disequilibrium of the c.3920T>A mutation with an allele at D5S346, which is 30–70 kilobases from the 3 prime end of the *APC* gene. [10]. This disequilibrium allows the determination of the precise *APC* gene allele lost.

### Extended PCR Analysis to Determine Other APC Somatic Mutations

Samples from the original DNA extractions from the colorectal tumours were prepared, and then analyzed independently by a second investigator (CT) to expand the analysis of the *APC* gene. The region of *APC* screened was in exon 15 and consisted of the mutation cluster region (MCR) and sequences 5′ of the MCR. This area encompasses coding nucleotides c.2147_4810, or genomic nucleotides g.112173438_112176101 (Build 36) and extends from codon 716 through codon 1604. The region was amplified using 26 pairs of primers. Reactions of 10 µl were carried out containing 2 ng/µl genomic DNA, 200 µM each dNTP, 1× Amplitaq buffer II (Applied Biosystems, Warrington, UK) http://europe.appliedbiosystems.com/), 2.5 mM MgCl2, 0.5 U Amplitaq Gold (Applied Biosystems), and 4 picomoles of each primer. Each pair of primers was labeled with either FAM or HEX. Cycling conditions were as follows: initial denaturation was at 94°C for 12 minutes followed by 36 cycles at 95°C for 30 seconds, annealing at 53–65°C for 30 seconds, and extension at 72°C for 30 seconds.

### Fluorescent single strand conformation polymorphism (FSSCP) analysis

PCR products were diluted 1/10 to 1/40 with water, depending on yield. An aliquot of 1 µl of diluted PCR product was mixed with 10 µl of formamide and 1 µl of ROX-500 size standards (Applied Biosystems), heated at 95°C for 2 minutes and snap-cooled on ice. The products were then subjected to capillary electrophoresis at 18°C and 30°C using a 3100 Genetic Analyser (Applied Biosystems), in 5% GeneScan polymer (Applied Biosystems,), 5% glycerol, 1× Tris-TAPS-EDTA buffer (Applied Biosystems) using 36 cm well-to-read capillaries. Data analysis was carried out using GeneScan and Genotyper software (Applied Biosystems) and by visual inspection of electropherograms.

### Extended DNA Sequencing

Samples in which a variant was identified by FSSCP were re-amplified using unlabeled PCR primers. PCR products were prepared for sequencing by treatment of 5 µl of PCR product with 2 U of shrimp alkaline phosphatase and 10 U of exonuclease I (Amersham Pharmacia, Chalfont St Giles, UK) at 37°C for 30 minutes and then 80°C for 15 minutes. Sequencing reactions of 10 µl were carried out using 1–3 µl of prepared PCR product, 1.6 picomoles of primer, BigDye ready reaction mix (Applied Biosystems) version 2 diluted 1∶2 with Half Big Dye reagent (Genetix, http://www.genetix.com/). Cycle sequencing conditions were 25 cycles of 96°C for 10 seconds, 50°C for 5 seconds and 60°C for 4 minutes. Unincorporated nucleotides and primers were removed by ethanol precipitation. Sequencing products were resuspended in 10 µl of formamide and run on 310 or 3100 genetic analysers (Applied Biosystems) using POP6 and 36 cm well-to-read capillaries. Data analysis was carried out by visual inspection of electropherograms.

### Statistical Methods

Analyses comparing the frequency of mutations by tumour type were conducted using contingency table analysis by either chi-squared test, or Fisher's exact test when the least expected value is less than 5. Unless otherwise stated, the *p* values result from chic square analysis [12].

## Results

A total of 432 patients was accrued to the studies of Ashkenazim. Fifty-seven (13.2%) individuals were carriers of the c.3920T>A germ line mutation. Thirty-seven (65%) of these carriers had two or more colorectal tumours, and from this group adequate DNA was available from tumours of 25 individuals. Tumour DNA from four additional carriers who had only one neoplasm was also selected. DNA from 82 tumours was therefore available from 29 patients; 27 were heterozygous carriers and two were homozygous for the c.3920T>A germ line variant.

These 29 patients were similar to the 28 patients whose tumours were not studied with respect to age at the time of removal of their first tumour, 61.8 years and 62.6 years respectively, (p = 0.74). The 29 patients whose tumours were studied had an average of 5.5 colorectal tumours removed over a period of 6.0 years. By contrast, the 28 patients whose tumours were not studied had just 2.3 tumours removed over 4.5 years (p</ = 0.0002 for number of tumors). Of the 29 patients studied, 20 (69%) were male and 9 (31%) were female. Of the 28 germ line carriers whose tumours were not studied, 18 (64.3%) were male and 10 (37.5%) were female.

The 29 persons studied had a total of 193 documented tumours up to the time of this study, consisting of 38 hyperplastic polyps, 121 tubular adenomas, 22 tubullovillous adenomas and 12 colorectal cancers. Pathology material was evaluated for 82 tumours (42.5%). We primarily studied the more advanced tumours on the assumption they would be more informative in terms of genetic changes. Forty-four (54.3%) tumours were removed from the right side of the colon and 37 (45.7%) were from the left side. Site was unknown for one tubular adenoma. Of these 82 tumours, 58 (70.7%) were tubular adenomas, 13 (15.9%) were tubulovillous adenomas, 9 (11.0%) were carcinomas and 2 (2.4%) were hyperplastic polyps. Genetic analyses were conducted in 2 separate laboratories (SM in NJ, USA; CT in Leeds England). In New Jersey, sequencing close to codon 1307 was conducted, as well as assessment for LOH; while in Leeds, more detailed sequencing analyses of exon 15 was conducted (see Methods). Results of these genetic analyses were compared for the overlapping region adjacent to codon 1307. Overall, there was strong correlation, with the few discrepancies resolved by reviewing the strength of evidence from each analysis.

### Germ line mutations of the APC gene

Sequence analyses confirmed the c.3920T>A variant in each tumour and in each normal sample. No other common variation was found in normal tissue, except for one person (3.4%) with both the c.3920T>A and the c.3949G>C germline variants. The latter reflects the missense E1317Q variant of no proven functional effect, as reported previously [13]. This patient had two tumours, a rectal cancer with no molecular change except for c.3924_3925insA, and a transverse tubular adenoma with LOH of the c.3920T>A allele. Two of our 29 patients were homozygous for c.3920T>A. One patient had three tubular adenomas; and just one, from the cecum, contained a nonsense mutation c.2677G>T. The second patient had two adenomas, tubular and tubulovillous, as well as a cecal carcinoma at age 71 years. The tubulovillous adenoma had a deletion c.4306delA, while the carcinoma had only a c.3924_3925insA.

### Somatic mutations of the *APC* gene

The c.3924_3925insA somatic mutation was the most frequent somatic change within the A8 tract, and it was found in 21 tumours and one hyperplastic polyp, or 22 of the 82 samples (26.8%) (Table 1). In 6 tumours, there was no evidence of remaining wild-type sequence,, and each of these tumors also showed LOH using the D5S346 microsatellite. In our previous study, by sub-cloning the APC region around codon 1307, we showed that the c.3924_3925insA somatic mutation invariably impacted the mutated allele.. In this analysis, comparison of the strength of the allelic ratio at codon 1307 with that at the site of c.3924_3925insA again demonstrated that the somatic mutation involving slippage of the A8 tract always occurred on the c.3920T>A allele. One tumour showing the c.3924_3925insA somatic mutation was homozygous for the germline c.3920T>A variant, and LOH could not be assessed.

**Table 1:** The number and percentage of tumours with somatic mutations in total and by anatomical location and pathology for all 82 tumours (excludes missense and silent mutations).

Tumour	Sample	Loss of	84648_84649	Other somatic mutations			Total # molecular changes		
Characteristics	size	heterozygosity*	insert A	None	One	Two	None	One	Two
	n	n (%)	n (%)	n (%)	n (%)	n (%)	n (%)	n (%)	n (%)
Total	82	19 (25.3)	22 (26.8)	48 (58.5)	30 (36.6)	4 (4.9)	25 (30.5)	36 (43.9)	21 (25.6)
Anatomical location									
Left colon	37	11 (29.7)	14 (37.8)	22 (59.4)	12 (32.4)	3 (8.1)	8 (21.6)	16 (43.2)	13 (35.1)
Right colon	44	8 (18.2)	8 (18.2)	26 (59.1)	17 (38.6)	1 (2.3)	17 (38.6)	19 (43.2)	8 (18.2)
Unknown site	1	0	0	0	1 (100)	0	0	1 (100)	0
Pathology									
Tubular adenoma	58	13 (22.4)	14 (24.1)	38 (65.5)	19 (32.8)	1 (1.7)	23 (39.6)	23 (39.6)	12 (20.7)
Tubulovil adenoma	13	3 (23.1)	3 (23.1)	4 (30.8)	8 (61.5)	1 (7.7)	1 (7.7)	8 (61.5)	4 (30.8)
Carcinoma	9	3 (33.3)	4 (44.4)	4 (44.4)	3 (33.3)	2 (22.2)	0	4 (44.4)	5 (55.6)
Hyperplastic polyp	2	0	1 (50)	2 (100)	0	0	1 (50)	1 (50)	0

Excludes 6 tumours from 2 patients homozygous for c.3920T>A.

**Figure 1:**
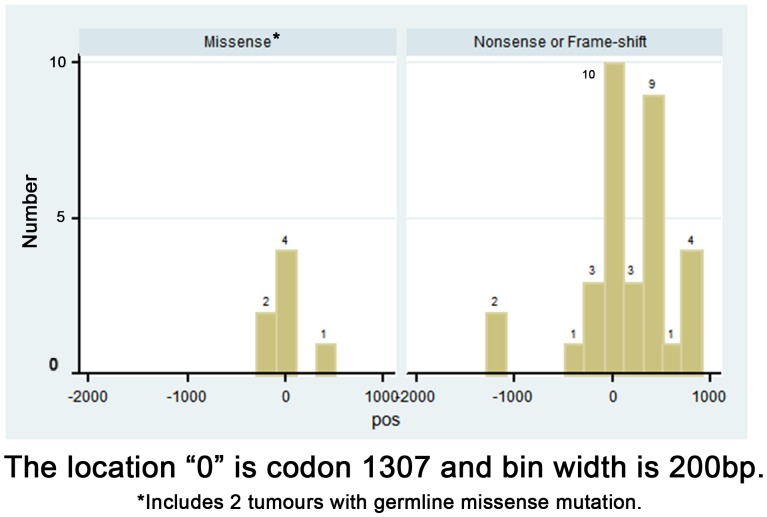
Distribution of location of somatic mutations by mutation type.

Other somatic changes were found in 38 of the 82 tumours (46.3%). A single somatic mutation was found in 31 tumors (37.8%), and 2 changes were found in 7 tumors (8.5%). Of these 45 somatic mutations, 38 were distinct. For the total, 21 somatic mutations were frame shift (46.7%), 17 nonsense (37.8%), 5 missense (11.1%), and 2 silent (4.4%) (see Table S1). For all 45 somatic changes, 9 (20%) occurred between codons 1296 and 1322, while 36 (80%) occurred outside this region. Figure 1 shows the location of the site of the somatic mutations by type of mutation relative to codon 1307. There were 7 missense mutations, of which 2 were germline, and all were found within 500 base pairs of codon 1307. Given the difficulty in determining the impact of these mutations on the function of *APC*, these remain of unknown disease significance. The nonsense or frame-shift mutations, which would result in a predicted smaller protein product, were far more numerous, with the majority being located 3′of codon 1307, with the nodal value at 1307.

A total of 75 of the 82 tumors were informative for LOH (Table 1). Six tumours from the two patients who were homozygous carriers of c.3920T>A were uninformative for LOH analyses because of complete linkage disequilibrium with D5S346. One additional tumour, a tubular adenoma, was not evaluated for LOH. A total of 19 tumours (25.3%) (16 adenomas and 3 cancers) demonstrated LOH. Loss of the wild type *APC* allele was detected in 13 tumours (68.4%) (12 adenomas, 1 cancer), and loss of the variant c.3920T>A allele was found in 6 tumours (31.6%) (Table 1). Eleven (29.7%) of the left-sided tumours demonstrated LOH as compared to 8 (18.2%) of the right-sided tumours, p = 0.29 (Table 1). Overall, there was no difference between the left and right sides of the colon for somatic mutations (p = 0.19, Fisher's exact test). There was no difference across pathologies for variation in LOH rates, but there were only 9 cancers and 13 tubullovillous adenomas, with the majority of tumours being tubular adenomas (58 tumors). Analyses of the c.3924_3925insA somatic mutation revealed limited variation across the pathology types. There were 3 (33%) cancers, 3 (23%) tubulovillous adenomas, and 34 (59%) tubular adenomas demonstrating no somatic changes other than c.3924_3925insA, (p = 0.04).

### Analysis of somatic changes

For the following results we ignore missense and silent *APC* mutations found in six tumours. Four tumours demonstrated a missense mutation, one had both a missense and a silent mutation, and one had just a silent mutation. Of these six tumours, three were tubular adenomas, two were tubulovillous adenomas and one was a hyperplastic polyp. Five of the six had other somatic changes; one tubulovillous adenoma had only the silent and missense mutations. For each of the other tumours, we have added the number of somatic events, counting 1 for LOH, 1 for the presence of the c3924_3925insA and 1 for each additional somatic mutation (nonsense or frame shift). Overall, 25 (30.5%) of the tumours showed no significant somatic molecular change, while 36 (43.9%) showed one change and 21 (25.6%) showed two or more molecular changes. Only one tumour (1%) showed more than 2 changes, demonstrating LOH of the wild-type allele, the c.3924_3925insA and a second A insert at c.4686_4687insA.

### Combinations of mutations

There was no association between the occurrence of c.3924_3925insA mutation and *APC* LOH for the 75 evaluable tumours, with p = 0.57 (excludes 6 tumours that were homozgyous and could not be evaluated for LOH, and one that was not evaluated for LOH). Of 21 tumours with the c3924_3925insA for which the LOH status was also known, 6 (29%) showed *APC* LOH and 15 (71%) did not. All six tumours with both the c.3924_3925insA and LOH demonstrated LOH of the wild type *APC* allele (p = 0.016), testing the one-sided hypothesis from previous observation [10]. No other somatic mutations were found in 5 of these 6 tumours. The sixth tumour had one somatic mutation, c.4686_4687insA. In the 15 tumours with the c.3924_3925insA somatic mutation and no LOH, 2 (13.3%) had a further single somatic mutation while 13 (86.7%) did not; the allele carrying the additional mutation is unknown (Table 2).

**Table 2:** Summary of pattern of mutations among the 75 tumours informative for presence of the c.3924_3925insA somatic mutation, loss of Heterozygosity (LOH) at APC and number of nonsense or frameshift mutations.

		Number of other somatic mutations		
Presence of c.3924_3925insA	LOH	None	One	Two
Yes	Yes	5	1	0
Yes	No	13	2	0
No	Yes	4	9	0
No	No	22	15	4

There was no significant difference in the prevalence of somatic mutations other than c.3924_3925insA in tumours with LOH as compared to those without LOH, with 10 of 19 tumours (53%) with *APC* LOH also demonstrating at least one somatic mutation, while 21 of 56 (37.5%) tumours without *APC* LOH demonstrated at least one somatic change, p = 0.29. Of the 19 tumors with LOH, 5 had the c.3924_3925insA mutation only, 9 had one other non- c.3924_3925insA somatic mutation, 4 had no other detectable mutation in the region, and one tumor had both the c.3924_3925insA change plus an additional insert. Six tumours showed LOH involving loss of the c.3920T>A allele; of these, 4 had a second somatic mutation while 2 had no other detectable mutation.

**Figure 2:**
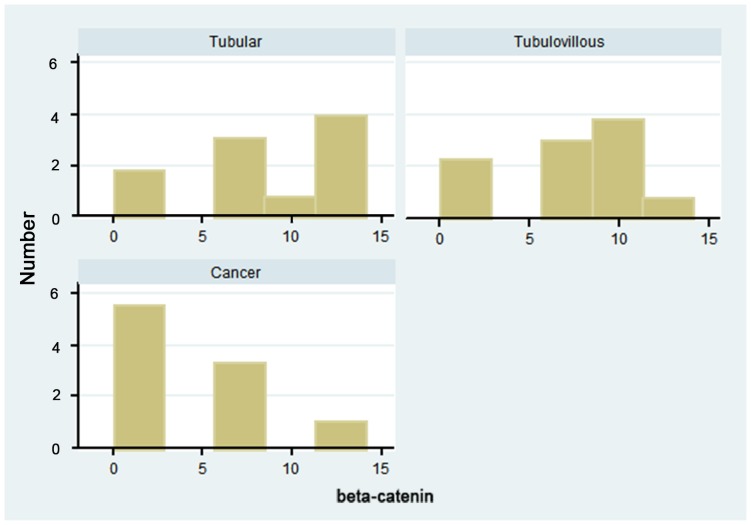
Number of beta-catenin repeats remaining by pathological type of neoplasm scored as 0–3 repeats, 4–6, 7–9, 10–12, 13–14.

The impact of mutations on the 20 amino acid beta-catenin binding repeats was considered. To conduct this analysis we assumed: (1) LOH is copy-neutral, and (2) the c.3924_3925insA always impacts the c.3920T>A allele. Wild type *APC* has 14 beta catenin-binding sites. For those tumours where mutations could not be phased, the maximum number of beta-catenin repeats remaining is shown (Figure 2). The pattern of repeats remaining shows similarity between tubular and tubulovillous tumours, with the majority of tumours retaining more than half of their repeats (69% for tubular and 77% for tubulovillous). However, as expected for cancers, the majority (67%) have less than 7 repeats remaining, although because of the limited number, the differences are not statistically different (p = 0.15, Fisher's exact test).

### Intra-person changes

Six of the 29 patients had only one tumour studied. Eighteen of the other 23 (78.3%) patients had no recurring molecular change in their neoplasms. These 18 patients had an average of 3.0 tumours studied, with a range from 1 to 5. Eighteen of the 23 patients had at least one tumour with an identified molecular change other than *APC* LOH or c.3924_3925insA in the region studied.

Five patients had identical somatic changes in more than one tumour, with the particular change absent from their normal tissue. The majority of one patient's tumours had a c.3924_3925insA at codon 1307 (4 tumors with the insertion [3 tubulovillous and 1 tubular] and one tubullovillous adenoma without the insertion). One patient had 2 tumours removed at different examinations from different anatomical locations, both with c.3991A>T mutation; one patient had 2 tumours removed at the same examination from the same anatomical location with c.4358delC; one patient had 2 tumours removed at the same examination from the same anatomical segment both with c.4393_4394delAG mutation; one patient had 3 tumours removed from the sigmoid colon but in separate examinations 8 years apart with c.4686_4687insA in all. Of the four patients with tumors demonstrating non-c.3924_3925insA repetitive somatic mutations, one patient had one of three tumors also with c.3924_3925insA. The other three patients demonstrated no c.3924_3925insA mutation in their tumours with repetitive changes. For these four patients, the incidence of c.3924_3925insA in their tumours with a repetitive somatic mutation is much less than a reported incidence of 26% [4].

## Discussion

Somatic mutations in the *APC* gene undergo selection, with combinations of mutations necessary for appropriate loss of function in colonic neoplasia [3]. In this study, we have investigated in detail the molecular genetics of colonic lesions from patients with the c.3920T>A germ line mutation. The goal was to assess in detail the impact of the c.3920T>A mutation and to screen for additional mutations - either germ line or somatic - in exon 15, from codons 716 to 1604. This region flanks the mutation cluster region located between codons 1280 and 1500. Twenty-five of the 29 patients had two or more tumours. We specifically chose patients with multiple tumours to increase the possibility of identifying other germ line mutations. In the original report of this germ line mutation, the authors reported that 40% of their tumours showed a somatic mutation in the vicinity of the germ line mutation as compared to 5% in a comparable series of non-c.3920T>A-derived tumours, indicating the notable local impact of this germ line variant [4]. For a more detailed analysis, we have expanded the region of the APC gene studied in these tumours.

The c.3920T>A mutation is a missense mutation involving the exchange of one amino acid for another. It is difficult to predict the consequences of missense mutations. Cleary et al. reported that germ line missense *APC* variants detected in patients with multiple colorectal neoplasms did not contribute to colorectal cancer risk in the general population [14]. Liang et al in a recent HuGE review reported a meta-analysis indicating a significantly increased risk for colorectal neoplasia (odds ratio of 2.17) for Ashkenazi Jewish carriers of the c.3920T>A variant [15]. The creation of the oligo-adenine tract (A8) by the c.3920T>A mutation is clearly highly associated with the insertion of an additional adenine (A9), occurring in 26% of the tumors analyzed for this paper, and the additional adenine produces a frame shift with definite functional consequences.

Our data suggest there is no significant contribution from other germ line *APC* mutations in the region studied to the phenotypic findings in our cohort. Of the 29 patients whose tumours were analysed, only one had an additional germ line mutation, c.3949G>C (p.Glu1317Gln), and this patient had only one tubular adenoma and a rectal cancer. Further, this patient's parents and children have all undergone colonoscopies and all were free of any colorectal tumours. The lack of any further common germ line variation suggests strongly that it is c.3920T>A which is the cause of the local genetic instability resulting in further somatic changes.

The clinical significance of the c.3949G>C mutation is questionable. Two early papers suggested a significant association with adenomas [16] and cancer [13], and subsequent analysis of the patients in the early cohorts showed that most cases could be accounted for by germline MUTYH mutations. A subsequent study of 538 Israeli colorectal cancer cases found no effect [17].

One study of young North American patients of various ethnic backgrounds with ten or fewer colorectal adenomas reported finding protein-truncating germ line variants within the *APC* open reading frame in 10 of 74 (13.5%) patients [18]. An indirect means of assessing the significance of the c.3920T>A mutation is to compare the phenotypic and genotypic differences between heterozygous and homozygous carriers. The phenotypic and genotypic findings for our two patients homozygous for c.3920T>A are quite similar to the findings for the 27 heterozygous patients. This suggests that the effects of c.3920T>A are quite weak.

LOH is not particularly useful in itself for assigning pathogenicity to a germ line mutation. However, it is expected to be a relatively frequent form of a'second hit'if the A8 tract of the c.3920T>A allele undergoes slippage. We found *APC* gene LOH in only 25% of tumours. The wild type allele was primarily lost, but one-third of the LOH was loss of the I1307K allele. This might suggest that for the development of many tumours, loss of the wild type allele is of no more benefit than loss of the c.3920T>A allele. We did not find somatic genetic changes to be related to site of tumour, type of tumour, or a combination thereof. We found the total number of somatic mutations occurring in a tumour to be equally likely as: “no mutations”, “one mutation “ or “2 or more mutations”.

A beta-catenin inhibitory domain (CID) of APC located between the second and third 20 amino acid repeats has been identified. The CID is a target of the selective pressure acting on APC during tumourigenesis, and it must be removed or inactiviated for the development of a colon carcinoma. This is more likely as additional 20 amino acid repeats are lost, and is reflected in our Figure 2 [19]. However, accumulated evidence suggests that retention of at least 1 to 2 of the 20 amino acid beta-catenin binding repeats in the truncated APC protein is usual, and that, paradoxically, this leads to lower levels of nuclear beta-catenin, and lower levels of Wnt signaling [20]. We have further considered our findings with respect to the number of beta-catenin binding sites remaining as a result of the various molecular changes detected. Our data suggest a correlation between advancing histology and fewer beta-catenin binding sites.

Five of our 29 patients had identical somatic changes in more than one tumour. For three patients, the repeating somatic change was distant from codon 1307, suggesting there is no major repeating influence from the APC*I1307K mutation among synchronous tumours. Two patients had several tumours with c.3924_3925insA mutations and a relatively severe phenotype. Some of their tumours had been evaluated only for *APC* LOH and c.3924_3925insA. One person had 5 tumours with this mutation and 5 tumours that did not. The other patient had 5 tumours with the mutation and 4 that did not. Interestingly, these lesions were distributed throughout the colon in both patients, suggesting that these do not represent partially excised lesions repeatedly examined. One potential explanation is that there remains an unidentified mechanism that increases the chance of this slippage. Supporting evidence for this is that the rate of LOH was high in the tumors with c.3924_3925insA, as 8 of the 10 (80%) also demonstrated LOH, which is higher than reported overall in the population, but similar to the frequency of LOH in FAP patients who carry the common codon 1309 mutation. However, no incriminating sequence variant was found in this analysis. The large number of tumours found in these two individuals suggests the possibility of a multiple polyp phenotype in addition to the presence of the c.3920T>A.

Identical mutations in multiple tumours from the same patient might reflect a real phenomenon, but statistically we cannot prove that the observations are more than chance, given the limited size of the sample set. If the pattern were observed in further tumours, and, as in this study, the tumours were sufficiently separated so as not to be clonal, then possible explanations include: 1] The presence of deleterious alleles elsewhere on chromosome 5 that mitigate against LOH in some cases; 2] local epigenetic modifications, such as cytosine methylation within *APC*; 3] an underlying tendency to a specific mutation type, for example, by relatively poor DNA repair or polymorphic fragile sites proximal to *APC*; 4] genetic or (micro)environmental modifiers of Wnt signaling that influence the optimal combination of *APC* mutations.

Our study has a few limitations. We evaluated a large area of exon 15, but we did not study other exons of the *APC* gene. However, previous studies suggest most *APC* gene mutations occur within exon 15 [21]. Furthermore, we did not simultaneously consider other genes known to be important in the development of colorectal tumours. Concurrent consideration of more than one gene would require a much larger sample size.

Our data represent a cross-sectional assessment of tumours, and, therefore, it is not possible to form firm conclusions regarding the sequence of molecular events. However, it does appear that for those tumours with the c.3924_3925insA and *APC* LOH, the c.3924_3925insA is on the mutated allele and LOH involves the wild-type allele. For other tumours in patients with c.3920T>A allele, it is possible that the c.3924_3925insA has not occurred before a somatic mutation develops on one or the other allele, followed by LOH. Alternatively, as in attenuated FAP, there could be selection for a third hit, i.e., loss of the c.3920T>A allele. Considerations regarding timing become additionally complicated for those tumors that are polyclonal [22].

We did not have a concurrent control group of similar tumours from Ashkenazi individuals without the c.3920T>A mutation, and this would be a worthwhile future effort. We found no notable characteristics of the mutations that we identified with the exception of c.3924_3925insA. Our main focus, however, was to assess for patterns within the group of tumours from patients with this particular germ line mutation. We did not study other exons of the *APC* gene, although exon 15 is a critical region. We also did not consider other genes known to be important in the development of colorectal tumours.

In conclusion, our data represent the most extensive evaluation of the *APC* gene in colorectal adenomas and carcinomas from Jewish carriers of the c.3920T>A mutation reported to date. We did not find any significant additional germ line mutations. The findings suggest the only somatic genetic change clearly attributable to the c.3920T>A mutation is the c.3924_3925insA. However, the finding that many tumours in our patients were not the result of A8 slippage at codon 1307 is consistent with the fact that c.3920T>A generally has only a modest effect on colorectal tumor risk.

## Supporting Information

Table S1 **This table indicates all Mutations detected and denotes the cDNA, Basic change, Amino acid change, and indicates the type of Mutation for all findings.**
(DOCX)
