# Supplementary figures and images for: Colorectal Tumors from APC*I1307K Carriers Principally Harbor Somatic APC Mutations outside the A8 Tract
Source: PLoS One. 2014 Jan 9;9(1):e84498. doi: 10.1371/journal.pone.0084498 (PMC3886998; doi:10.1371/journal.pone.0084498)

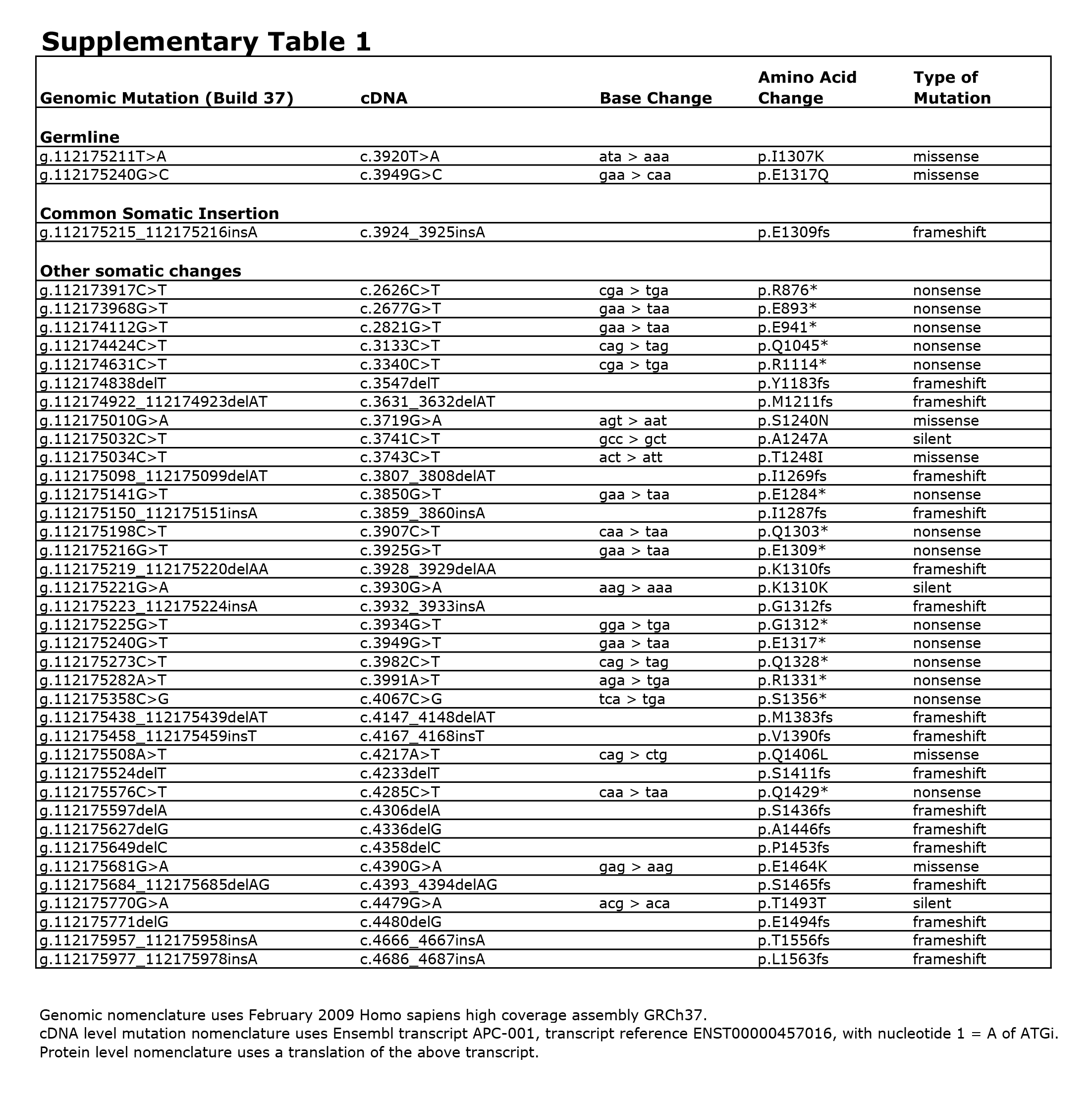

Supplement: Table S1 — This table indicates all Mutations detected and denotes the cDNA, Basic change, Amino acid change, and indicates the type of Mutation for all findings. (DOCX) [file pone.0084498.s001.docx]
